# Hospital and Institutional News

**Published:** 1914-08-29

**Authors:** 


### HOSPITAL AND INSTITUTIONAL NEWS.

### OUR EDUCATIONAL NUMBER.

Next week, as usual, The Hospital will publish a Special Educational Number, giving information, not merely of the curricula at the various hospital medical schools and universities, and of interest to students, but also a survey of the profession, of interest also to fully qualified men. In spite of the war, the various universities, as our correspondence columns show, are endeavouring, so far as possible, to continue their ordinary courses. But it would be idle from a general standpoint to suppose that the war will have no influence on the profession at this time. A special article, therefore, will deal with "The Effect of the War on Professional Prospects," and the opportunities afforded by the Army and Navy Medical Services, now inevitably engaging the attention of medical men and students, will be specifically dealt with. In addition, we shall include the chief points of war news from our special correspondents, in so far as it relates to hospitals, and the usual features of the paper will be included, so far as space allows. The price of the Special Number will be, as usual, one penny.

### AN ENGLISH BASE HOSPITAL FOR FRANCE.

The French Government has been offered a base hospital containing 200 beds for the wounded of the allied forces, and the offer has been accepted by the French Ambassador. Approval has also been expressed by the War Office, and the suggestion thus welcomed emanates from an English committee of which the Duchess of Roxburghe is the president, Sir Owen Philipps chairman, and Sir Starr Jameson, M.D., vice-chairman. Thanks to the War Office's approval, the transport of the hospital's equipment will be expedited. The hospital itself will be under the French Red Cross. The surgeon-commandant is Mr. Ernest Miles, and the object of the base hospital and its staff is to treat on the spot serious cases which are not in a condition to be moved to England. The staff has been engaged, and the expedition is starting this week. Sir Owen Philipps, who is a Knight of Grace of St. John of Jerusalem in England, and was accorded the Grand Cross of the Spanish Red Cross in 1912, has been a member of the executive committee of King Edward's Hospital Fund since 1908. From his close connection with the Elder, Dempster, and other shipping interests, it is also interesting to recall that he holds the post of vice-president of the Liverpool School of Tropical Medicine. Sir Starr Jameson, who graduated in medicine in 1877, well known in connection with the raid on the Transvaal of 1895, became director of De Beers in 1900, and was Premier of Cape Colony from 1904 to 1908.

### HOSPITAL SECRETARIES ON ACTIVE SERVICE.

In addition to Mr. Thomas, secretary of the Hampstead General and North-West London Hospital, who, as we have already noted, was called up to join the Honourable Artillery Company early this month, Mr. Allen Naldrett, secretary of the Royal Southern Hospital, Liverpool, has also left for active service. As a lieutenant in the Royal Army Medical Corps (T.) he quickly joined his unit, and is now attached to the 1st Western General Hospital at Fazakerley, Liverpool. His work is being carried on, we understand, by Mr. John S. Birbeck, the senior member of his secretarial staff. We also understand that Mr. Reginald B. Jay, secretary of the Royal Sussex County Hospital, Brighton, is with his Territorial regiment, and that Mr. Louis McCausland, secretary of the Kensington and Fulham General Hospital, who is a sergt.-major in the National Reserve, is assisting in the mobilisation of the local battalion under Col. Simpson, commanding officer. We shall be glad to receive news of any other hospital secretaries thus actively engaged with the Forces.

### PUBLIC PHARMACISTS AT THE FRONT.

It is gratifying to notice that an increasing number of public pharmacists and dispensers are volunteering to assist in the national emergency. Mr. C. H. Hampshire, B.Sc., pharmaceutist at the University College Hospital, and until recently one of the teaching staff at the Pharmaceutical Society's School of Pharmacy, has joined the colours as a member of the Honourable Artillery Company. Mr. Montagu Smith, F.C.S., dispenser to the Lewisham Board of Guardians, has been allowed to join the 5th Battalion Royal West Kent Regiment, and his board undertake to keep his post open whilst he is on active service. Some disappointment has been expressed that the War Office authorities have not seen fit to recognise the training and education of pharmacists by offering commissions.

### INFIRMARY SUPERINTENDENTS AND SCHEMES OF PUBLIC WORK.

The Local Government Board has issued a circular letter to the County Councils and Urban District Councils suggesting steps that should be taken in order to mitigate the distress which will arise from unemployment during the war. In this letter the Board says: "In other cases the distress committee in co-operation with the local authority will probably be able to initiate schemes of work by which provision could be made for the more deserving and necessitous cases. Such schemes will be aided by grants made by the Board out of the money provided by Parliament for the purposes of the Unemployed Workmen Act. In London, the Central (Unemployed) Body has circularised Boards of Guardians, asking for particulars of any schemes the Guardians could put in hand. This is a matter which should receive the careful consideration of the medical superintendents of our Poor-Law infirmaries. Structural alterations and additions are needed in many instances. Some of the London infirmaries, for instance, do not possess operating theatres; in others improvements in the wards and their annexes and the provision of rooms for *x*-ray work or the examination of clinical material are needed. Schemes of this character should be brought to the notice of the Boards of Guardians, so that applications for grants of assistance could be made. Work of this kind is eminently work of public utility, and the possibility of obtaining monetary aid for its performance is an argument which will appeal strongly to many boards, especially in the poorer districts.

### THE WAR'S EFFECT ON PANEL PRACTICE.

The effect of the war on the treatment of insured persons is causing some anxiety, and leading to a variety of stopgap arrangements. In Birmingham, for instance, the Insurance Committee, noting that fourteen doctors were already on service, are trying to find substitutes in cases where the panel practitioners have not arranged for locum tenentes to take their place. The work in other cases of doctors who have been called up is being shared out among other practitioners. Precautions are also being taken in the matter of drugs, especially those of which no further supply is expected from Germany. In Dundee the position seems more serious. No fewer than sixteen panel doctors are affected by the calling out of the Reserve and Territorial Forces, affecting, it is said, 25,000 insured persons. The local medical committee have, therefore, submitted a scheme to the Insurance Committee, which has approved it. It provides for the establishment of a central office and a central dispensary, for which the out-patient department of the infirmary will be used. All messages from patients where doctors are absent will be sent to the central office, and visits will be distributed by locality and by rota among doctors willing to take part in the scheme. In even normal times the medical service in Dundee is proportionately less numerous than elsewhere in Scotland, and the war has, of course, increased the shortage. The scheme, therefore, embodies private as well as panel practice, and, except in the records, no discrimination is to be made between the two.

### A CORP OF MASSEUSES FOR ACTIVE SERVICE.

The War Office is announced to have accepted a scheme by which a corps of skilled masseuses are being enrolled to serve anywhere where required. The scheme is being financed by Mr. Almeric Paget, the borough member of Parliament for Cambridge, who proposes to allow £2 a week to all full-time masseuses on active service, to cover board and lodging, with a grant of £3 for uniform. Dr. F. Barrie Lambert is the medical officer in charge of the corps, and Mr. Essex French is the organising secretary. Applications for enrolment in the "Almeric Paget Massage Corps" can be obtained from, and should be returned to, Miss French, care of Mr. Almeric Paget, M.P., St. Stephen's Chambers, Westminster. It may sound surprising that such a corps exists for purposes of war, but the acceptance by the War Office of the offer shows that those best qualified are satisfied, as was averred at the time, that by the services of skilled masseuses during the Balkan War the wounded were saved three or four weeks in hospital.

### THE QUEEN'S NAVAL HOSPITAL, SOUTHEND.

As we noted in our issue of August 15 (page 548), an appeal is being issued by the Duke of Portland to support the Royal Naval Hospital at Southend-on-Sea, of which the Queen has accepted the post of president. Thanks to Mr. Alfred Tolhurst, the Palace Hotel has been converted into a hospital, and, what is more, has been approved of by Fleet-Surgeon Mundy after an inspection, on behalf of the authorities. In addition to previous information as to staff and accommodation, we may add that being on the sea cliff the hospital should be very easily within reach of those disembarking the wounded from hospital ships, while its site, at Southend, is a central one for the arrival of ambulance vessels from the Navy and the Continent. The improvised operating theatre is a large, light room with walls almost entirely of glass. These facts are of interest in view of the advice which the number of offers of private hospitals has led the Red Cross Society to urge, that, namely, no hospital should be actually prepared before a mobilisation order has been received from the military authorities. The Society also urges that funds should not be prematurely laid out.

### CONVALESCENTS AND HOME DISTRICTS.

As we foresaw a week or two ago, the place of the convalescent home in this country is assuming increased importance. The Queen has now suggested that sick soldiers and sailors should be sent to convalescent homes in their home districts. This small but valuable proof of sympathy should not require much additional organisation to carry out, and the Medical Directors-General of the War Office and the Admiralty have been consulted and expressed their sympathy with the plan. To carry it out a small committee has been formed and will make preliminary inquiries, with a view to laying before the Medical Directors-General information as to the best way of acting on the Queen's suggestion.

### A MONASTERY AS A HOSPITAL.

The Isle of Wight is unwontedly busy. Its existence as a pleasure resort beside the Solent is now modified by the active preparations which are being made in readiness for any sick and wounded who may be landed there. At the Isle of Wight County Hospital, Ryde, the medical staff have been given a free hand with reference to the admission of wounded soldiers. But of more curious interest is the fact that the monks of Quarr Abbey—so well known to visitors to the island—are converting the greater part of their monastery into a hospital for the wounded, and are maintaining it at their own expense. There are few groups of "alien immigrants" (we use the term in no offensive sense) capable of such organised charitable effort, but, if the reports from Paris of the enlistment of many priests be correct, the Church is taking a very active part in this war, and the share of the monks of Quarr Abbey will be remembered gratefully to their credit, provided that expert assistance and medical supervision are given to the hospital which they have kindly improvised. We should be glad to have assurance on this point from the monks themselves, or hospital men in their neighbourhood.

### SOME PANEL DOCTORS' GRIEVANCES.

At a meeting of the West Sussex Insurance Committee at Worthing last week a report was presented by the Medical Benefit Sub-Committee stating that panel practitioners had not yet received any payment for unallocated insured persons, in spite of the fact that agreements were signed on the understanding that this should be done. The local medical committee considered this was a breach of faith, and desired that further inquiries should be made as to the doctors' mileage fund. The non-settlement of this matter is also causing much discontent. The Committee decided to reply that a complete settlement of the panel fund was imminent, and that a scheme in respect of the mileage fund was under consideration and would be submitted for the approval of the local medical committee. Moreover, the clerk in a report on the revised credit in respect of medical benefit, pointed out that there was a discrepancy of over 3,000 insured persons between the figures on which the Commissioners had based their calculations and those ascertained from the Committee's index register. It was decided to call the Commissioners' attention to this, and to point out that unless a more equitable scheme was drawn up for future credits the Committee could not fairly discharge its obligations to the doctors.

### OFFICIAL ATTITUDE TO AUXILIARY HOME HOSPITALS.

The British Red Cross Society is informed by the military authorities that the time has not arrived when definite assurance of the necessity of utilising proffered houses as temporary hospitals can be given. The various offers are being codified and arranged with regard to the counties in which they are situated, and their relation to the various military hospitals. Offers from public institutions are being placed on a special list, and those fully staffed and equipped have been particularly noted as likely to be useful. Temporary hospitals to be established by Voluntary Aid Detachments form an important link in the Territorial military organisation. No Voluntary Aid Detachment will, it is stated in a memorandum on the subject issued by the Red Cross Society, be utilised except in the district in which it is registered, although the military authorities may ask for volunteers for duty elsewhere. "At present," the circular proceeds, "Voluntary Aid Detachments are asked not to prepare any actual hospitals, but to have schemes for them ready to open at any moment, when required." Such hospitals belong to the category of Temporary Hospitals, and their equipment, except in the case of a small supply of drugs and dressings, should not necessarily consist of articles except such as can be obtained from neighbouring houses. We quote the final words of the memorandum in full: "It is most important therefore—(1) That no hospital should be actually prepared before the mobilisation order of that hospital has been received from the military authority, in order that *personnel* and equipment which may be urgently needed elsewhere shall not be uselessly locked up. (2) That funds should not be prematurely laid out on the preparation of these hospitals which might be more usefully expended in other directions. (3) That the work of institutions intended for other purposes, such as schools and colleges, must not be interfered with, except on military requisition or in consultation with the educational authorities. (4) That Voluntary Aid Detachments have no authority whatever to take over of themselves any public or private building."

### SCHOOL BUILDINGS AS MILITARY HOSPITALS.

The Board of Education has sent a circular to local education authorities, in reply to their numerous requests for advice in respect of the demands of Voluntary Aid Detachments for the use of school buildings as military hospitals. The Board state that the Director-General of the Army Medical Service informs them that Voluntary Aid Detachments are entitled to make use of public educational buildings only on specific instructions from the officer commanding the district. The Director-General adds that no emergency is likely to arise in the near future requiring the occupation of school buildings other than those already requisitioned. This announcement should do something to stop the excessive anticipation of actual hospital requirements which has been going on during the last fortnight.

### THE WELSH WAR HOSPITAL SCHEME.

On the suggestion of Dr. James Robinson, the Lord Mayor of Cardiff, a Welsh war hospital of 100 beds for the Army has been offered to the War Office. The cost is expected to be £15,000, and it is hoped that when complete it will accompany the Expeditionary Force wherever that may be serving. Sir W. J. Thomas has given £2,500. Lieut.-Col. A. W. Sheen, R.A.M.C., who was in charge of the Welsh hospital in South Africa, has been appointed commanding officer. In supporting the proposal at the public meeting which endorsed it, Col. E. M. Bruce Vaughan, chairman of King Edward VII.'s Hospital, Cardiff, said that that institution would offer 150 beds, and they would also have the assistance of the Red Cross nurses and the St. John Ambulance Association.

### THE TRANSFORMATION OF CAMBRIDGE.

As Cambridge is one of the most important Red Cross hospital centres in the Eastern Counties, the following bird's-eye view of its transformation from a peaceful university to a town in military occupation is of interest. Cambridge is the 1st Eastern Hospital. A very large number of troops, variously estimated, have arrived in Cambridge from different parts of the country, and their destination is not known. The troops commenced to arrive a fortnight ago, and have continued to pour in since. They are encamped in and around Cambridge, and a very hearty welcome has been extended to them on all sides. Gifts of fruit, refreshment, tobacco, papers, magazines, and books have not been lacking. Several private tents have been erected in the various camps where the troops may obtain fruit, light refreshment, etc., at lowest possible charges, and the work in connection with these has been undertaken by a number of patriotic ladies and gentlemen. Father Waggett, the well-known Cowley Father, has placed the greater part of his spacious residence in Chilston Road at the disposal of the soldiers as reading and writing rooms. These are but a very few instances of the way in which the residents of Cambridge are welcoming our troops. It will be recalled that it was Sir Alfred Keogh, when Director-General of the Army Medical Service, who established hospitals for the Territorial branch of the Service, which previously had no medical organisation of its own. Cambridge is one of the twenty-three general hospitals, each possessing 520 beds and a local staff of trained medical men and nurses, which his scheme established, and for which we must all be so grateful to-day.

### COMPLAINTS AGAINST A WANDSWORTH INFIRMARY.

The Wandsworth Board of Guardians have held an inquiry into the complaints referred to in The Hospital of August 1 (page 483) regarding the treatment of patients in St. James' Hill Infirmary, Wandsworth. The matter was brought to the notice of the Guardians by the Mayor of Battersea, who stated that the mother of a boy had removed him from the infirmary, owing to the treatment he received. It was alleged that he had been allowed to lie unprotected from the sun on the verandah until his face was badly scorched; that his food was served in an unsatisfactory state; and that there were only one sister and four nurses in charge of seventy-five patients. An inquiry, lasting two hours, was held by the Infirmary Committee, and they came to the conclusion that they could find no just cause for the complaint. When this report was presented to the Board at the last meeting it led to an acrimonious discussion, and was only adopted by twelve votes to ten, and even those who voted in favour of its adoption had to acknowledge that the institution was seriously understaffed. The Mayor of Battersea pointed out that with only four nurses and a sister to seventy-five patients, either the nurses are overworked or the patients are not sufficiently looked after. The Acting Chairman, Mr. Percival Rees, said they all knew there was a scarcity of nurses, and that the number of nurses at this infirmary was only what the Local Government Board would allow them, but this was a matter that would have the attention of the committee. The inquiry has had at least one good effect, for it has drawn attention to the understaffing at the infirmary, and it is hoped the Guardians will immediately press the Local Government Board to grant them a substantial increase.

### THE NAMING OF POOR-LAW INFIRMARY WARDS.

The Hull Guardians had under discussion at their last meeting the naming of the wards in their new hospital. The hospital committee suggested the wards should be named after those members who had passed through the chair whilst the hospital had been under consideration and erection. After a lively discussion, the Guardians rejected the proposal by one vote. In our opinion the majority is to be commended for its decision. The wards of most Poor-Law infirmaries are denoted by letters and numbers. This is not an ideal arrangement and some infirmaries have improved upon it by naming their wards after the more important virtues. Again, a few infirmaries commemorate in their ward names the lives of men and women worthy of national or local honour. Such a method is to be praised, but the names chosen should be of those most deserving to be held in memory by their townsmen. They should not be of such formality as will be meaningless in a few years' time.

### THE PRIVACY OF SANATORIA PATIENTS.

At last week's meeting of the Halifax Health Insurance Committee, over which Mr. A. Culpan, the chairman, presided, the Sanatorium Sub-Committee presented a curious minute. In this it was reported that "female insured patients receiving treatment at the Shelf Sanatorium had expressed a desire to leave the institution owing to the lack of privacy there." It was then pointed out by Mr. T. N. Kaye that the male and female wards were divided by wide doors which, it was complained, were constantly kept open in such a way that patients in each ward could see one another getting out of bed and dressing. Dr. Branson, chairman of the Halifax Corporation Health Committee, said he had told the matron that the complaint was reasonable, and that "a screen between the wards would obviate all difficulty." We can hardly agree that this is so; the arrangement and construction of the wards must be faulty which allow them to be open to each other's view in this way, and which has to rely on a screen for mutual privacy. Such makeshifts are administratively bad, and give rise to trouble, and we prophesy to Dr. Branson that though the point may seem small, it is a small thing due to an administratively faulty principle, and as such will give rise to endless trouble if it is not dealt with. If two wards are in such immediate proximity as to be virtually one and the same, one sex only should be accommodated in them.

### THE BURNLEY DISPUTE.

A temporary and perhaps a permanent settlement of the long dispute between the Burnley Guardians and their District Medical Officers is likely to be reached as a result of the war. Last month the Guardians offered as a basis of amicable settlement to reinstate the six public vaccinators subject to the four following points being agreed to:—(*a*) That all medical officerships become vacant at an early date. (*b*) That during the interregnum the relieving officers issue medical tickets to the sick and needy on the nearest doctor. (c) That a further conference be held between representatives of the doctors and the Guardians to consider the readjustment of the proposed twelve districts and the salaries attached thereto. (d) That advertisements should then be issued for twelve medical officers for the twelve proposed districts. This proposal was submitted to the local division of the British Medical Association and was rejected on the ground that no proposals for settlement could be entertained until the public vaccinators who were dismissed, and who were in no way concerned in the original dispute, had been reinstated. Within the last few days, however, the division passed the following resolution and forwarded it to the Guardians:—"That in view of the present crisis, should the Board of Guardians feel that they are unable to cope with the Poor-Law medical relief, the Burnley committee of the British Medical Association will have no objection to the late district medical officers making arrangements through this committee with the Guardians for attendance on the deserving poor during the period covered by the war." The reading of this communication was received with cheers at the last meeting of the Guardians and was referred to the special committee which has the matter in hand.

### THE UNOCCUPIED WARDS AT WORCESTER INFIRMARY.

At the recent monthly meeting of the Executive Committee the difficulties created by the war were discussed, and among them the Matron's statement that during the high prices of food the nurses and servants behaved most reasonably, and denied themselves in bacon, butter, and other expensive articles of food. Lady Hindlip wrote on behalf of the Worcester district of the Worcestershire Branch of the British Red Cross Society, asking, by request of the Voluntary Aid Detachments of Worcester, if the Committee would consent to lend the now unoccupied wards, with furniture and equipment, the use of a kitchen, sleeping accommodation for the nurses, and occasional use, if necessary, of the operating theatre, for sick and wounded, the control to be taken over by their Commandant. The Chairman said he had replied that they had already offered the use of two wards to the War Office, and that they had not yet had any reply, but that they could not do anything in the matter until they heard from the War Office. The medical men and nurses on the staff had consented to tend cases of sick and wounded in the war free. The Dean said that to hand over control of part of the building to another body would cause endless confusion. The Chairman also said that they could not consent to a dual control. Mr. Byrne pointed out that additional nurses under the control of the Committee would be welcome. The Chairman remarked that they were very short of nurses, and some were going on active service. The Matron had said that honorary probationers would be extremely useful. It is curious to note that, as we predicted, the policy of closing wards is not only unfortunate in itself, but in the present condition of affairs is actually creating greater difficulties.

### THE COMBINED WAR SCHEME AT BRISTOL.

The arrangements for establishing Bristol as a base hospital centre are moving rapidly, and, in addition to the Royal Infirmary, the Red Cross Society has received the offer from the Board of Guardians of the new Southmead Infirmary—the buildings are nearing completion, and it was hoped would be ready for occupation in October or November. The combination of the above two institutions will largely facilitate the centralising of the medical and nursing staffs, while the Southmead Infirmary, which is practically a hospital and convalescent home in one, will furnish valuable aid. The Thornbury Board of Guardians are willing to devote their new infirmary of sixty beds to the Red Cross Society if necessary, but this has for the time being not been accepted.

### INSTITUTIONAL PHARMACISTS AND ARMY RANK.

Few, if any, of the pharmacists of our leading hospitals appear to be attached, in their professional capacity, to the Army Medical Service. Indeed, a correspondent suggests that "institutional pharmacists would appear to have a distinct grievance in that, if they wish to become 'dispensers' in the R.A.M.C., they must enlist unconditionally as 'privates,' and are not accorded any rank befitting their professional training, consequently 'dispensers'—to use which title no statutory qualification is required—are chiefly recruited from the ranks of chemists' assistants and senior apprentices." Although the present is hardly an opportune time to press for an official rank to be accorded pharmacists, yet it is a matter which should receive the earnest consideration of the Army authorities when the present war is over.

### FIFTY YEARS OF HOSPITAL DISPENSING.

The retirement is now taking place of Mr. J. T. Kilner, who for over fifty years has been dispenser at the Huddersfield Royal Infirmary. Beginning his connection with the institution as junior dispenser, Mr. Kilner after three years was promoted to the senior post, and for fifty-two years has been head of his department. On behalf of the board Dr. Marshall recently presented him with a cheque for £100, to which generous members of the board and of the honorary staff and subscribers had contributed. His period of service probably constituted a record, and they had never subscribed to a testimonial with greater pleasure. A special resolution of thanks has also been passed by the governors, and Mr. Kilner will enter on his retirement with a record behind him not only of exceptional length, but also of exceptional good will. He must have seen many changes in institutional dispensing, on which he did not dilate in reply to the presentation. The evolution of the dispenser into the pharmacist and the growth of proprietary medicine, with its effect on prescribing, are two factors which indicate how great the change has been. With the vistas opened up by the Insurance Act he may well think his fifty years of previous work enough to entitle him to a well-deserved retirement.

### POOR-LAW INFIRMARIES AND THEIR DRUG CONTRACTORS.

The supply of drugs, referred to in the last issue of The Hospital, page 565, has exercised the consideration of a number of the Metropolitan Boards of Guardians. Notifications have been sent out that the contractors are unable either to supply goods at the contract prices or are unable to supply them altogether. This applies more especially to drugs of German manufacture, but, as in the case of the Lambeth Board of Guardians, the medical staff and the dispensers have been able to find suitable substitutes. It is true that the tender forms do not contain a clause exempting them from operation during a time of war, but there are other questions to consider at a crisis like this than the bare cut-and-dried words of an ordinary contract form. The point is a highly debatable one as to whether a contract signed in a period of peace would hold good in a court of law during a time of war. To do so, it must be fair and equitable, and it is doubtful whether any judge would hold that the terms of such a contract were fair and equitable under existing conditions. Many Guardians hold the opinion that the contracts must stand, but in London the reverse opinion has been expressed, and the Guardians have acted on the advice of their clerks. It must be remembered that the matter cuts both ways. The contractor loses on his contract maybe, but the Guardians are bound to find drugs and food for those in their infirmaries, and, as the money comes out of the rates, they will feel more lenient to the contractors. This spirit has been generally shown by Boards of Guardians who have intimated to their contractors that they will be ready at the close of their contracts to consider reasonably any extra expenditure incurred by the contractors.

### A GENERAL DRUG STORE FOR LONDON.

There is another solution to this difficulty which might well be considered when it is remembered that next month the great majority of drug contracts to Poor-Law institutions expire. It is evident that the Guardians will experience extreme difficulty in getting firms to tender for a twelve- or even six-months' supply of drugs, and under these circumstances they might well consider the advisability of having one large general drug store for the whole of London, from which the various institutions might draw their requisite supplies. At some institutions there is a supply of drugs sufficient to last a month, and in a few isolated cases only two months. Hence the need at the present time for a policy of conciliation on both sides. What are our readers' opinions?

### "HEATHER DAY" IN MANXLAND.

Normally at this season thousands of persons go to enjoy a holiday in the Isle of Man. Glasgow Fair holidays brought recently 15,000 holiday-makers in one week-end, and to these an appeal was made on behalf of Noble's General Hospital, the only one of any size on the island, which is situated in beautiful surroundings at Douglas. The Scotch residents evolved the idea of making up button-holes composed of heather and tartan ribbons and selling these to their countrymen on holiday. The Speaker of the Manx Parliament was at the head of affairs, assisted by an energetic committee of ladies and gentlemen; and their efforts were rewarded by collecting the fine sum of £200 in one day. It is hoped to make this "Heather Day" a yearly institution, and with organisation on bigger lines it will be an added help to carry on the work. Other holiday resorts might follow the Manx example, and they are already being urged that there is at present no need for panic and no necessity for ordinary visitors to be turned away.

### A CORPS FOR CHOLERA SERVICE.

We are informed that a scheme is on foot to organise a special corps for cholera service with the Russian forces, and the Russian Embassy is waiting to hear from the Russian Red Cross on the point. The scheme at present contemplates the enrolment of ten medical men and twenty nurses. Medical volunteers for this work should have had practical experience of cholera as clinicians or bacteriologists, and nurses should have had a full hospital training. Applications, in the first instance by letter only, may be sent to the Secretary of the Provisional Committee, 67 Margaret Street, W. Dr. A. White Robertson is responsible for the proposal, and has associated with him Dr. Dorothea M. Tudor, M.B., B.S., who had experience during the Balkan War.

### THE WAR AND THE PRICE OF DRUGS.

Considering the abnormal conditions under which business is being transacted, the state of the Drug market must be regarded as satisfactory. Attempts to make purchases in unnecessarily large quantities have ceased, and, with the exception of the chemical products made in Germany, stocks of drugs are sufficient to last for a sufficient length of time while supplies continue to be received from over the seas. Very much higher prices are, of course, quoted for many drugs. For instance, acetyl-salicylic acid is nearly four times the price quoted before the war; carbolic acid is at least double the price; potassium bromide and other bromides have more than doubled in value; chloral hydrate is three times the price; citric acid has nearly doubled in value as also has cocaine; quotations for lithia salts have also been nearly doubled, and the same applies to phenacetin and saccharin. Acetanilide, gallic acid, tannic acid, bismuth salts, iodides, antipyrine, pilocarpine, and sulphonal are among the other drugs which have advanced in value very considerably, while thymol is among the drugs which are practically unobtainable. There seems no prospect that any of these drugs will be reduced in price under present conditions, and all that can be done is to cover immediate requirements.

